# Supplementary material for: Exploration of a GMMA-Based Bivalent Vaccine Against Klebsiella pneumoniae
Source: Vaccines (Basel). 2025 Feb 24;13(3):226. doi: 10.3390/vaccines13030226 (PMC11946371; doi:10.3390/vaccines13030226)
Supplement: Supplementary file 1 [file vaccines-13-00226-s001.zip › vaccines-3433681-supplementary.pdf]

Supplementary Tables

Table S1. The bacterial strains and plasmids used in this study.

Strains /Plasmids		Relevant characteristics	Source
Klebsiella Pneumoniae Strains	KP-O1	O1 serotype *Klebsiella pneumoniae*	Fourth Medical Center of the People's Liberation Army General Hospital
	KP-O2	O2 serotype *Klebsiella pneumoniae*	Fourth Medical Center of the People's Liberation Army General Hospital
Plasmids	KP-GMMA	KP-304-2*ΔtolRΔpagPΔlpxM*	This study
	pUC19_CRISPR-DpmrA	*Klebsiella pneumoniae* mutant plasmid	Anne-Catrin Uhlemann's laboratory[1]
	pKP-CRISPR	*Klebsiella pneumoniae* mutant plasmid	This study
	pACYC184-KPO1	O1 polysaccharide expression plasmid	Li Zhu's laboratory[2]

Table S2. List of primers and their sequences used for construction of *K. pneumoniae* deletion mutant strains.

Primer	sgRNA Sequence (5’- 3’)	Identification of Sequence (5’- 3’)
pagP-F	ACGGACCAGAATTTCCACCT	AATTTGCTGGGTTCGTTTGCG
pagP-R	ACGGACCAGAATTTCCACCT	ATGGGTCATATAGGTGGCGAA
tolR-F	GAGACTTCCACAATCACCGG	GGCTTCCTGCGCTTCTTTTG
tolR-R	GAGACTTCCACAATCACCGG	GCTGAAGCGTTCGAGGACAA

lpxM-F	ACGATACGACCATCGCCCGG	CTTTAATCCGCGTCGTCTGAAC
lpxM-R	ACGATACGACCATCGCCCGG	CCCAGATGATGGAGTCGATGATC
plasmidB-1-F	AGTTCAGCTCCAGCTCCCAG	TGGCCAGGGTACAAGCTTAT
plasmidB-1-R	AGTTCAGCTCCAGCTCCCAG	AGCCCAGTCAGACTTTGCTG
plasmidB-2-F	GGAAGCGGGATACTGCACGG	CACTTTTCGGGGAAATGTGC
plasmidB-2-R	GGAAGCGGGATACTGCACGG	GCAAGATTGGTATCCTTGGC
plasmidE-1-F	ATCGAACTGGATCTCAACAG	CAGCGCAACGGAACATTCAT
plasmidE-1-R	ATCGAACTGGATCTCAACAG	TGCCGTCAATCCCGACTTCT
plasmidB-2-F	AGAATAGGTAGTGAACTGGG	GCCATTACCTGCAGACTGAG
plasmidE-2-R	AGAATAGGTAGTGAACTGGG	TGGTATGCCGGCATACAGCA

Table S3. Identification of top 20 proteins information by LC-MS/MS.

Sample	Protein IDs	Description	Unique peptides	Sequence coverage	calc. pI	emPAI
WT-OMV	A0A0H3GU43	Murein lipoprotein	5	53.8	8.87	2510.886
	A0A0H3GMF7	Outer membrane protein A	25	82.1	5.8	447.925
	A0A0H3GVB4	ATP synthase subunit beta	26	85.9	5	382.119
	A0A0H3GHX9	60 kDa chaperonin	34	76.5	4.94	375.494
	A0A0H3GSR0	Uncharacterized protein	8	70.3	8.62	315.228
	A0A0H3GNL7	Glycoprotein/polysaccharide metabolism	2	27.0	7.44	214.443
	A0A0H3GUI9	Outer membrane protein X	9	54.1	8.72	165.81
	A0A0H3GQC1	Peptidoglycan-associated protein	8	68.4	6.8	157.489
	A0A0H3GMR2	Putative porin	7	59.1	6.95	128.155

	A0A0H3GNC0	Acetyltransferase component of pyruvate dehydrogenase complex	29	64.4	5.31	105.421
GMMA	A0A0H3GU43	Murein lipoprotein	7	60.3	8.87	25117.86
	A0A0H3GQC1	Peptidoglycan-associated protein	10	71.8	6.8	1994.262
	A0A0H3GMF7	Putative outer membrane lipoprotein	24	82.4	5.8	903.736
	A0A0H3GUH2	Uncharacterized protein	9	77.4	8.87	420.697
	A0A0H3GSR0	Outer membrane protein X	8	70.3	8.62	397.107
	A0A0H3GUI9	Outer membrane protein W	10	72.9	8.72	358.381
	A0A0H3GS09	Putative porin	8	52.7	5.57	358.381
	A0A0H3GMR2	Glycoprotein/polysaccharide metabolism	7	59.1	6.95	214.443
	A0A0H3GNL7	Penicillin-binding protein activator LpoB	2	27.0	7.44	214.443
	A0A0H3GVJ1	Putative homeobox protein	7	58.0	8.88	198.526

Supplementary Figures

Figure S1

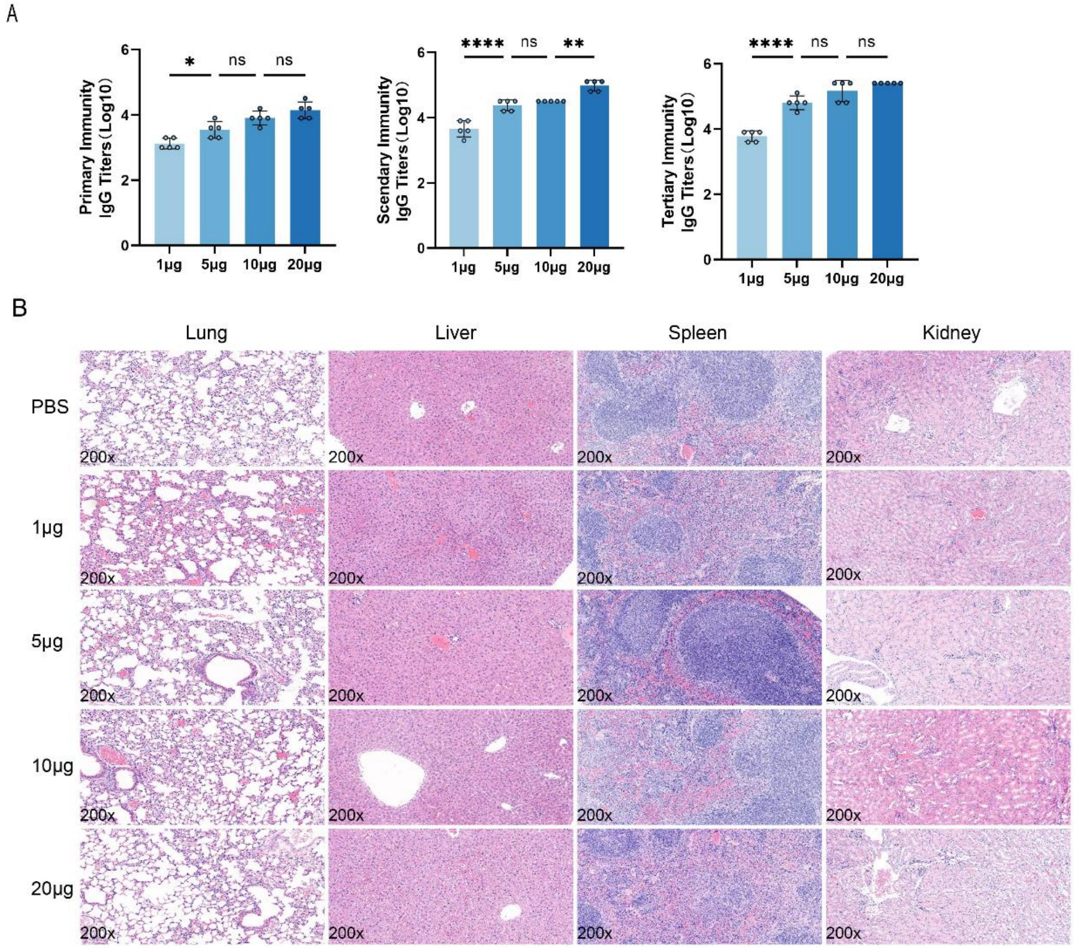

Figure S1 Immunogenicity and safety of GMMA with different immune doses in mice. (A) ELISA analysis of IgG antibody titers induced by varying dosesof GMMA following a single administration (left), two administrations (middle), and three administrations (right). (B) Pathological changes in tissues of mice immunized with various doses of GMMA. The immune doses were set as 1, 5, 10, or 20 μg/animal successively. Differences between groups were tested using one-way ANOVA. *, $p<0.05$, **, $p<0.01$, ****, $p<0.0001$, ns $p > 0.05$.

Reference

1. McConville, T.H.; Giddins, M.J.; Uhlemann, A.-C. An Efficient and Versatile CRISPR-Cas9 System for Genetic Manipulation of Multi-Drug Resistant Klebsiella Pneumoniae. *STAR Protoc.* **2021**, *2*, 100373, doi:10.1016/j.xpro.2021.100373.
2. Liu, Y.; Pan, C.; Wang, K.; Guo, Y.; Sun, Y.; Li, X.; Sun, P.; Wu, J.; Wang, H.; Zhu, L. Preparation of a Klebsiella Pneumoniae Conjugate Nanovaccine Using Glycol-Engineered Escherichia Coli. *Microb. Cell Factories* **2023**, *22*, 95, doi:10.1186/s12934-023-02099-x.
